# Air pollution and health – the importance of air monitoring and burden of disease for attaining SDGs

**DOI:** 10.1093/eurpub/ckac129.018

**Published:** 2022-10-25

**Authors:** C Martins

**Affiliations:** 1 NOVA National School of Public Health, Lisbon, Portugal; 2 Comprehensive Health Research Center, Lisbon, Portugal

## Abstract

Pollution is the worldwide largest environmental cause of disease and premature death, being also considered a risk for planetary health, a cause of ecosystems destruction and intimately linked to global climate change. Pollution is considered costly, with attributable diseases resulting in health-care costs that are responsible for 1.7% of annual health spending in high-income countries. Moreover, in utero and early childhood exposures are responsible for health effects in children. The importance of air pollution as a cause of disease is well reflected in the Sustainable Development Goals (SDG), mainly in the SDG 3, SDG 11, and SDG 15, but indirectly in all the SDGs. To tackle this issue, an integrative and holistic approach linking human and environmental health such as One Health is needed, to provide evidence-based data to support the establishment of reduced air pollutants’ maximum admissible levels. A case-study developed in Portugal in the scope of PMCardImpact project, regarding the exposure of population to particulate matter with a diameter of 2.5 μm or less (PM2.5) and the associated number of cases of cardiovascular diseases will be presented herewith. Four scenarios of exposure will be considered for presenting the results: current scenario of exposure, new WHO Air Quality guidelines, European Commission Air Quality Directive and lastly, a worst-case scenario. This assessment is the starting point for calculation of the burden of disease of CVD that exposure to PM2.5 represent in Portugal. With a view to promote the science to policy interface, PMCardImpact project will make available to policy makers the needed supporting information to act, including actionable knowledge on air pollution trends and related health effects, to implement reducing air pollution policies.

This work is funded by FCT/MCTES through national funds to PMCardImpact (EXPL/SAU-PUB/0944/2021) and CESAM (UIDP/50017/2020 + UIDB/50017/2020 + LA/P/0094/2020).

